# Isolation and evaluation of a *Bacillus altitudinis* strain to improve cigar tobacco leaves fermentation

**DOI:** 10.3389/fbioe.2025.1636506

**Published:** 2025-08-12

**Authors:** Hejun Liu, Xin Fang, Shiping Guo, Bo Zhang, Chongde Wu, Qiu Zhong, Ruina Zhang, Hongzhi Shi, Yanqing Qin, Shuhua Zeng, Yao Jin

**Affiliations:** ^1^ College of Biomass Science and Engineering, Sichuan University, Chengdu, China; ^2^ Key Laboratory of Leather Chemistry and Engineering, Ministry of Education, Sichuan University, Chengdu, China; ^3^ R&D Department, Sichuan Provincial Branch of China National Tobacco Crop Tobacco Science Institute, Chengdu, China; ^4^ Deyang Tobacco Company of Sichuan Province, Deyang, Sichuan, China; ^5^ College of Tobacco Science, Henan Agricultural University, Zhengzhou, China; ^6^ College of Agronomy, Sichuan Agricultural University, Chengdu, China

**Keywords:** tobacco cigar leaves, fermentation, microbial community, functional strain screening, bioaugmentation

## Abstract

**Introduction:**

High levels of nitrogenous compounds such as proteins and alkaloids in cigar tobacco leaves can negatively impact combustion, smoke smoothness, and overall sensory quality. At the same time, the formation of aroma-active compounds during fermentation is essential for desirable flavor development. This study aimed to isolate a functional bacterial strain capable of accelerating nitrogen compound degradation and enhancing aroma quality through bioaugmented fermentation.

**Methods:**

A total of 65 bacterial strains were isolated from naturally fermented cigar tobacco using a tobacco extract-based medium. Sixteen showed significant protease activity, and *Bacillus altitudinis* (CCTCC M20211370) was selected for further study. Laboratory- and industrial-scale fermentation trials were conducted with this strain. Volatile compounds were analyzed using gas chromatography–mass spectrometry (GC-MS), microbial community dynamics were assessed via high-throughput sequencing, and sensory evaluations were performed to assess quality improvements.

**Results:**

Inoculation with *Bacillus altitudinis* significantly increased desirable aroma compounds such as neophytadiene and β-ionone, while reducing harmful compounds including nicotine and myosmine. The microbial structure was reshaped, with enrichment of beneficial genera like *Bacillus* and *Oceanobacillus*. Sensory analysis confirmed improved aroma complexity, featuring enhanced floral, honey-sweet, and resinous notes.

**Discussion:**

*Bacillus altitudinis* effectively improved cigar tobacco quality by promoting nitrogen compound degradation and modulating microbial metabolism to enhance aroma development. These findings support its potential as a bioaugmentation agent in industrial cigar fermentation. Further genomic and enzymatic studies are warranted to elucidate its functional mechanisms and facilitate large-scale application.

## 1 Introduction

Tobacco, one of the most widely cultivated and consumed non-food crops globally, is commercially available in diverse forms (Liu T. et al., 2022). Among them, cigars are particularly prized for their distinctive flavor. As a significant commercial tobacco product, cigars enjoy widespread popularity in North America and internationally (Vilela et al., 2020), undergoing drying, fermentation, and aging processes (Jensen and Parmele, 1950).

Cigar tobacco’s upper leaves contain macromolecules such as starch and proteins, with a low sugar-to-nitrogen ratio. High starch content negatively affects the combustion properties of the tobacco, while excessive protein leads to unpleasant odors and irritants during combustion. High pectin content can result in undesirable woody and off-flavors in the smoke (Liu F. et al., 2022). Additionally, the total alkaloid content, total sugars, potassium levels, and the alkali-to-nitrogen ratio all influence the aroma profile, aftertaste, and irritancy of the tobacco. Water-soluble sugars play a crucial role in neutralizing acidic and alkaline smoke compounds during combustion, reducing smoke irritation (Liu F. et al., 2022; Wu Q. et al., 2023) and acting as important precursors for tobacco aroma (Chen et al., 2019). Therefore, fermentation is a key treatment for enhancing both combustion properties and flavor characteristics of the tobacco.

Cigar tobacco fermentation is critical to its final quality and flavor. Through complex microbial metabolic activities, macromolecules like proteins and starch are broken down into smaller molecules, leading to the formation of aromatic compounds (Shibin et al., 2021). However, traditional fermentation processes suffer from long cycles, low efficiency, and inconsistent flavor stability, limiting the large-scale production of high-quality cigars. Recent advancements have focused on the use of functional microorganisms to enhance fermentation and regulate tobacco metabolism, offering promising solutions for improving cigar quality. By selectively screening microorganisms with specific metabolic functions, fermentation cycles can be shortened, and flavor compound production enhanced (Zhang et al., 2024). Therefore, exploring the roles of functional microorganisms in cigar tobacco fermentation and analyzing their metabolic mechanisms hold great theoretical and practical value (Wen et al., 2024).

Previous studies have shown that excessive nitrogen-containing compounds are a major cause of off-flavors in cigar smoke, making their reduction crucial for improving cigar quality (Chunpin, 2015). The microbial community on cigar tobacco leaves (CTLs) is diverse, with genera such as *Bacillus*, *Actinobacteria*, and *Pseudomonas* playing key roles in fermentation (Liu T. et al., 2022; Ning et al., 2023). However, most existing studies focus on microbial diversity, and systematic research on the targeted screening of functional microorganisms and their metabolic regulation mechanisms remains limited (Zhang et al., 2023). The relationships between microorganisms and volatile aroma compounds are not well understood, which hinders the precise application of functional microorganisms in fermentation processes (Liu et al., 2021; Wu X. et al., 2023).

Therefore, this study isolated several core functional microorganisms with protein-degrading abilities from high-quality CTLs. A strain of *Bacillus altitudinis* (CCTCC NO: M 20211370) was chosen to serve as the bioaugmentation agent for the fermentation process of cigar tobacco. The effect of such strain on volatile compounds, microbial structural of tobacco fermentation were then evaluated.

This study systematically reveals the functional characteristics of *Bacillus altitudinis* in cigar tobacco fermentation, providing a theoretical and technical foundation for microbial community regulation and fermentation optimization. The findings not only expand the application of functional microorganisms in the tobacco industry but also propose new research directions for developing bioaugmentation strategies for high-quality fermentation industry.

## 2 Materials and methods

### 2.1 Tobacco samples and culture conditions

The cigar tobacco samples, consisting of naturally fermented high-grade second-class tobacco leaves including wrapper, binder, and filler types, were from Shifang, provided by the Sichuan Provincial Branch of China National Tobacco Crop Tobacco Science Institute.

Tobacco extract was prepared using fermented cigar tobacco from Shifang as raw material. The tobacco leaves were finely ground and then subjected to a 40-mesh sieve to ensure a uniform particle size. In the preparation process, 15 g of the resulting tobacco powder was combined with 400 mL of distilled water. This mixture was then heated to a boil and allowed to extract for a duration of 30 min. After boiling, the extract was filtered through two layers of gauze to remove any solid residues. To preserve the integrity of the extract for subsequent applications, it was cooled and stored at a temperature of 4 °C.

Protein-hydrolyzing microorganisms were cultured and isolated, and protease activity was assayed under specified conditions: tobacco extract (TE) medium (100 mL L^-1^ tobacco leaf aqueous extract, 900 mL L^-1^ distilled water, 4.5 g L^-1^ yeast extract, 9.0 g L^-1^ tryptone, 9.0 g L^-1^ NaCl, with natural pH), fermentation medium (5.0 g L^-1^ cigar tobacco leaf powder and 20.0 g L^-1^ glucose), casein medium (10.0 g L^-1^ casein, 4.0 g L^-1^ NaCl, 5.0 g L^-1^ yeast extract and 20.0 g L^-1^ agar powder, adjusted to pH 7.2). All the chemicals and reagents used were of analytical grade.

### 2.2 Isolation and purification of microorganisms from tobacco leaf surfaces

Exactly 1 g of high-quality fermented tobacco leaf sample was accurately weighed and finely chopped using sterile scissors under aseptic conditions. The shredded tobacco was immersed in 100 mL of sterile water and allowed to incubate at 37 °C while shaking at 200 rpm for 30 min. Following the incubation period, the mixture underwent filtration and was then centrifuged at 9,000 × g for 15 min. The resulting supernatant was removed, and the pellet was reconstituted in 5 mL of sterile water to obtain the initial bacterial suspension.

The original bacterial suspension was serially diluted (10^–1^ to 10^–5^), and 100 μL of every dilution was uniformly spread on TE agar. After incubating the plates upside down at 37 °C for 48 h, bacterial colonies became visible. Single colonies were purified by quadrant streaking until pure isolates were obtained.

### 2.3 Directed screening of functional microorganisms

Single colonies were inoculated into casein agar and incubated at 37 °C in an inverted position for 48 h. Strains with faster growth and larger casein hydrolysis zones were selected for subsequent purification. The selected isolates were pretreated using the glutaraldehyde fixation method (Feng et al., 2019) for scanning electron microscopy observation with a SU8010 microscope (Hitachi High-Technologies, Japan) at a magnification of ×10,000 , using secondary electron signals with a working distance (WD) of 9.9 mm.

Genomic DNA extraction was carried out utilizing a commercially available DNA extraction kit. The identification of bacteria was achieved through PCR amplification targeting the 16S rDNA region employing universal primers 27F (5′-AGTTTGATCMTGGCTCAG-3′) and 1492R (5′-GGT​TAC​CTT​GTT​ACG​ACT-3′), with products generated using the 1×TSE101 Gold Mix from Qingke Biotechnology Co., Ltd. (China), resulting in amplicons of about 1,500 bp. The assembled amplified sequences were processed through ContigExpress and subsequently uploaded to the NCBI database. For sequence alignment, the Basic Local Alignment Search Tool was utilized, and a phylogenetic tree was created employing the neighbor-joining method in accordance with the Kimura two-parameter model, including 1,000 bootstrap replicates (Shin et al., 2014; Sakulworakan et al., 2021). The reagents and equipment used for sequencing were supplied by Shanghai Meiji Biomedical Technology Co., Ltd. (Shanghai, China).

### 2.4 Optimization of enhanced fermentation conditions

Functional microorganisms isolated and purified in this study were streaked onto solid media to obtain single colonies, which were subsequently inoculated into liquid fermentation medium and incubated at 37 °C for 48 h to prepare the initial bacterial suspension. Bacterial cells were harvested by centrifuged at 8,000 × g for 10 min, resuspended in sterile physiological saline, and adjusted to a final concentration of approximately 1.0 × 10^9^ CFU mL^-1^ to prepare the stock inoculum. An equal volume of cryoprotectant solution containing 10% (v/v) glycerol was added, and the mixture was thoroughly homogenized, aliquoted into sterile cryovials, and stored at −80 °C. Prior to use in fermentation experiments, the inoculum was revived from frozen stocks and cultured in liquid medium until reaching a concentration of 3.82 × 10^8^ CFU mL^-1^ for application. The fermentation process was conducted at 37 °C with a relative humidity of 70% over a period of 10 days, using a seed liquid application rate of 100 mL per kilogram of tobacco material. Three experimental groups were established to evaluate fermentation effects under different treatments: an enhanced fermentation group (GD), in which seed liquid was added; a blank control group (KB), which received the same volume of distilled water instead of seed liquid; and a natural fermentation group (ZR), which was left untreated without any additives or interventions.

### 2.5 Microbial community structure

Microbial community analysis of CTLs from different fermentation treatments was conducted using triplicate biological replicates. Genomic DNA was extracted from homogenized cigar tobacco samples (40-mesh particle size). DNA purity was assessed using a NanoDrop 2000 spectrophotometer (Shoeb et al., 2017), and DNA integrity was verified via 1% agarose gel electrophoresis.

To analyze bacterial community composition, the V3–V4 region of the 16S rRNA gene was amplified using universal primers 338F (5′-ACT​CCT​ACG​GGA​GGC​AGC​AG-3′) and 806R (5′-GGACTACHVGGGTWTCTAAT-3′) (Penland et al., 2020). For fungal diversity analysis, the ITS1 region was amplified using primers ITS1F (5′-CTT​GGT​CAT​TTA​GAG​GAA​GTA​A-3′) and ITS2R (5′-GCT​GCG​TTC​TTC​ATC​GAT​GC-3′) (Rimington et al., 2018). The PCR products underwent gel purification and were quantified with a Quantus™ fluorometer (Promega, United States).

Purified amplicons were combined in equal molar concentrations and then processed through paired-end sequencing (2 × 300 bp) on the Illumina MiSeq PE300 platform (Illumina, San Diego, CA, United States). The initial sequencing reads underwent quality filtering with fastp (version 0.20.0) (Chen et al., 2018) and were subsequently merged using FLASH (version 1.2.7) (Magoč and Salzberg, 2011). OTUs were established at 97% sequence similarity (Edgar, 2013) using UPARSE (version 7.1) (Stackebrandt and Goebel, 1994), while chimeric sequences were eliminated. The taxonomic classification was carried out with the RDP Classifier (version 2.2) against the SILVA database for bacteria (16S rRNA) and the UNITE database for fungi (ITS), utilizing a confidence threshold of 70%. The raw sequencing data has been submitted to the NCBI SRA database, with the BioProject accession number PRJNA824254.

### 2.6 Determination of volatile flavor compounds

The analysis of volatile metabolites from fermented CTLs was conducted after a 7-day fermentation period utilizing a GC-MS system (7890B/5977A, Agilent Technologies, United States) outfitted with a HS-SPME apparatus. An exact weight of 0.5 g of finely ground tobacco was combined with 10 μL of ethyl phenylacetate (chromatographic grade) serving as the internal standard. The samples were subjected to equilibrium at 70°C for 20 min, followed by extraction for 30 min. Finally, qualitative and quantitative analysis of the aroma components was performed using the NIST mass spectral library and Agilent ChemStation.

### 2.7 Sensory quality evaluation

To further validate the fermentation capability of *Bacillus altitudinis*, we conducted an industrial-scale fermentation verification experiment with a 1500 kg batch. The CTLs used for sensory evaluation were sourced from this industrial verification experiment. The fermented CTLs was rolled into cigars measuring 120 mm in length and 15 mm in diameter. The cigars were conditioned at 20°C and 60% relative humidity to achieve moisture equilibrium. Subsequently, sensory quality evaluation was conducted by seven trained panelists specializing in cigar production and assessment, following the standardized evaluation form provided by the Changcheng Cigar Factory (Jia et al., 2023). Sensory analysis was performed based on two main aspects: quality characteristics and flavor attributes. For quality characteristics, eleven parameters (e.g., richness, maturity, and irritation) were rated on a 0–9 scale, with higher scores indicating better performance. For flavor attributes (e.g., floral, roasted, and honey-sweet notes) were rated on a 1–5 scale, with higher scores denoted greater intensity. Consensus on evaluation scores for each sample was achieved among all panel members.

### 2.8 Analytical methods

Proteolytic activity was assessed by casein hydrolysis zone analysis (Shen et al., 2021). The diameter of the bacterial colony (d) and the diameter of the hydrolysis (clear) zone (D) were measured using a ruler. The proteolytic index (I) was calculated to evaluate the strain’s protein degradation ability Equation 1.

I=D/d (1)

Strains with strong protein hydrolysis ability were inoculated into tobacco extract liquid medium and cultured at 37 °C until the optical density at 660 nm (OD_660_) reached approximately 1.0.

Protease Activity Assay: A crude enzyme solution of 5 mL was subjected to centrifugation at 8,000 × g for 5 min at a temperature of 4 °C. The resulting precipitate was removed, and the supernatant was combined with 50 mL of phosphate buffer, followed by appropriate dilution to create the enzyme test solution. The measurement of protease activity was conducted using UV spectrophotometry at a wavelength of 660 nm with a spectrophotometer (Model L7, INESA Scientific Instrument Co., Ltd., Shanghai, China).

Total nitrogen, protein, starch, reducing sugars, and total sugars were quantified using a continuous flow analysis system (San++Classic, Skalar, Netherlands). The methods for total nitrogen determination followed GB 5009.33–2016, protein content was assessed as per YC/T 166–2003, starch levels were measured according to GB 5009.9–2016, and reducing sugars were evaluated using the GB/T 5513–2019 standard. For each sample, three biological replicates were performed.

### 2.9 Statistical methods

Principal coordinate analysis (PCoA) was utilized to assess variations in microbial communities among cigar samples originating from various fermentation groups, employing the Bray–Curtis distance matrix for both dimensionality reduction and visualization. The relative quantities of metabolites were determined through a semi-quantitative peak area normalization technique. Compounds that exhibited similarity index (SI) and reverse similarity index (RSI) scores exceeding 750 were chosen for further investigation. The relationships between volatile metabolites and microbial communities post *Bacillus altitudinis*-enhanced fermentation were illustrated with heatmaps generated using R software (version 4.1.2) (Hoyles et al., 2018). Functional annotation along with pathway analysis for genes and enzymes was executed based on the KEGG database. In examining the correlations between volatile metabolites and microbial communities, a significance level of p < 0.05 (n = 3) was established for evaluating statistical significance.

## 3 Results

### 3.1 Strain isolation and identification

Previous studies have shown that the protein content of the macromolecular substances in naturally fermented CTLs is associated with the flavor quality of the tobacco (Wen et al., 2023). A high protein content can lead to poor smoking characteristics, producing unpleasant odors and off-flavors (Song et al., 2024). Therefore, elimination of such off-flavors in CTLs requires a reduction in the protein content, which can improve the smoking quality of cigars. In this study, we focused on selecting microorganisms with the ability to degrade the macromolecular protein substances in tobacco leaves, using protein degradation as a screening principle. The microorganisms were then preserved, identified, and subjected to further fermentation performance testing.

**FIGURE 1:**
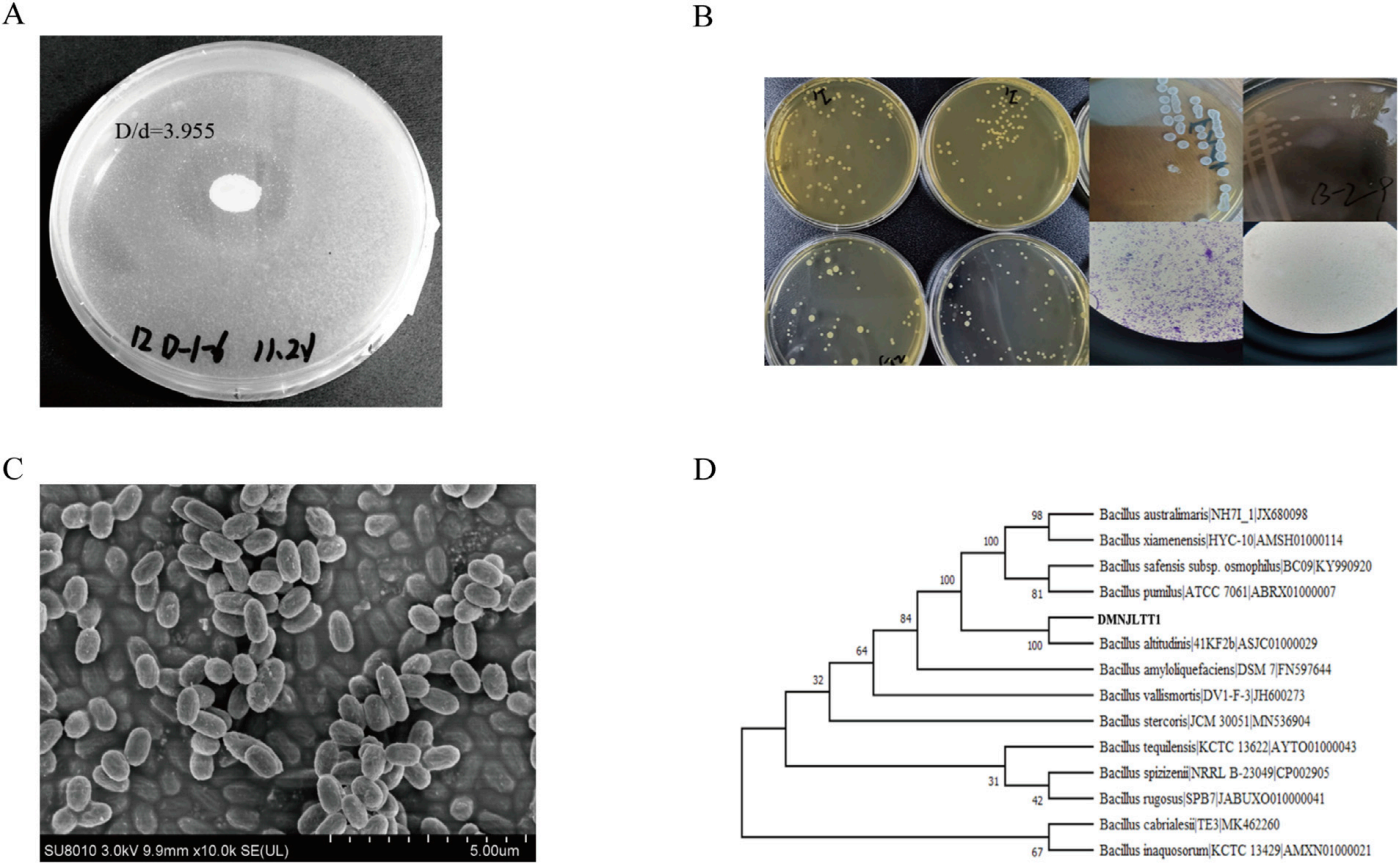
Screening and identification of functional microorganisms. **(A)** The experimental results of casein hydrolysis by *Bacillus altitudinis* strain; **(B)** Morphological observation and Gram staining of strains; **(C)** Scanning electron microscopy results of the strain; **(D)** Phylogenetic tree of strain *Bacillus altitudinis*.

A total of 65 bacterial isolates were obtained through gradient dilution plate method. Given the key regulatory role of protein metabolism in the fermentation process, protein hydrolysis ability was established as the core screening criterion. 16 strains producing protease were selected based on casein hydrolysis circle analysis. Among them, the strain (CCTCC NO: M 20211370) exhibited outstanding degradation efficiency, with a hydrolysis index (D/d value) of 3.955 (Figure 1. A). Further quantitative enzyme activity assays confirmed that its protease activity peak reached 577.15 U mL^-1^ (Supplementary Figure S1), establishing it as the preferred strain for subsequent fermentation enhancement studies. The colonies of the strain appeared round, opaque, and slightly raised, with rough or irregular edges and a dry to matte surface texture. Gram staining indicated that the strain is Gram-positive (Figure 1. B). Scanning electron microscopy revealed that the cells exhibited a rod-shaped morphology (1.2–2.5 μm) with a rough surface and aggregated arrangement (Figure 1. C). Phylogenetic analysis showed that the strain is a Gram-positive *Bacillus*, displaying 100% sequence identity with *Bacillus altitudinis* (Figure 1. D). The strain has been preserved in a −80 °C glycerol stock for further study.

### 3.2 Comparative analysis of volatile compounds

**FIGURE 2:**
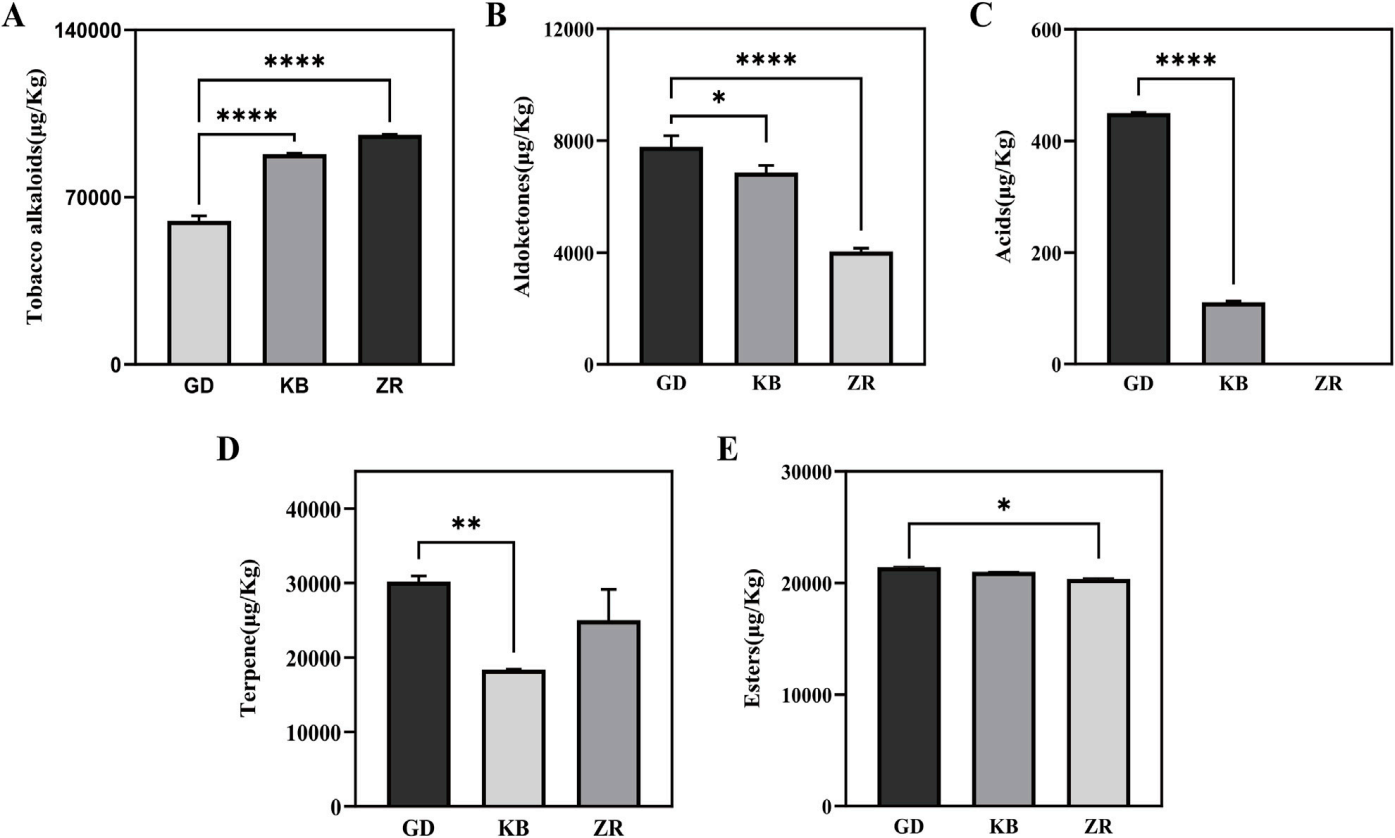
Total volatile substances in cigar tobacco of Shifang binder leaf by *Bacillus altitudinis* enhanced fermentation. **(A)** Tobacco alkaloids; **(B)** Aldoketones; **(C)** Acids; **(D)** Terpene; **(E)** Esters.

A total of 21 volatile metabolites were identified from cigar tobacco samples, including three tobacco alkaloids, five terpenoids, five esters, six aldehydes and ketones, and two acids (Supplementary Table S1). Compared to the other two groups, the GD group exhibited a significant alteration in the composition of volatile flavor compounds: the content of tobacco alkaloids decreased by 31.6%, aldehydes and ketones increased by 13.4%, terpenoids increased by 64.3%, acids Dramatically increased and small increases were also observed in esters (Figure 2).

In terms of aroma-contributing substances, the GD group presented 10 additional compounds compared to the ZR and KB groups, including plant-derived furans, ethyl palmitate, linalool, geranylgeraniol, (±)-3-hydroxylauric acid, and citrus ketone. Moreover, levels of solanone, geranylacetone, neophytadiene, β-selinene, and cedrene were elevated, while geranylacetone slightly decreased. Furans produced by Maillard reactions are known to contribute caramel-like aromas (Seeman et al., 2003; Song et al., 2024). The increase in aldehydes and ketones is favorable for flavor enhancement due to the presence of carbonyl groups, which are key aroma-active moieties and impart pleasant fragrances (Lei et al., 2015). Solanone contributes to the smoothness of tobacco aroma (Hong-qi, 2005; Teng, 2013), ionone adds sweetness (Wang et al., 2022), and decanal provides a fresh, citrus-like scent (Zhang et al., 2022). Terpenoids are important aromatic components in tobacco, such as neophytadiene and cedrene (Wang et al., 2024).

These results suggest that *Bacillus altitudinis* not only degraded nitrogen-containing compounds such as tobacco alkaloids, but also activated additional metabolic pathways, thereby increasing both the quantity and diversity of terpenoids and carbonyl compounds. This activity is likely to contribute positively to quality improvement and aroma enhancement during cigar fermentation.

### 3.3 Comparative analysis on microbial structure

Microbial community diversity was analyzed across the three groups of fermented cigar tobacco leaf samples. A total of 430,590 optimized bacterial sequences and 179,283,491 fungal sequences were obtained, with an average sequence length of 416 bp. Taxonomic classification revealed: 1 domain, 1 kingdom, 11 phyla, 18 classes, 42 orders, 63 families, 95 genera, 121 species, and 142 OTUs. The GD group exhibited a decreasing trend in bacterial diversity and richness, while fungal diversity showed no significant difference (Supplementary Figure S2).

**FIGURE 3:**
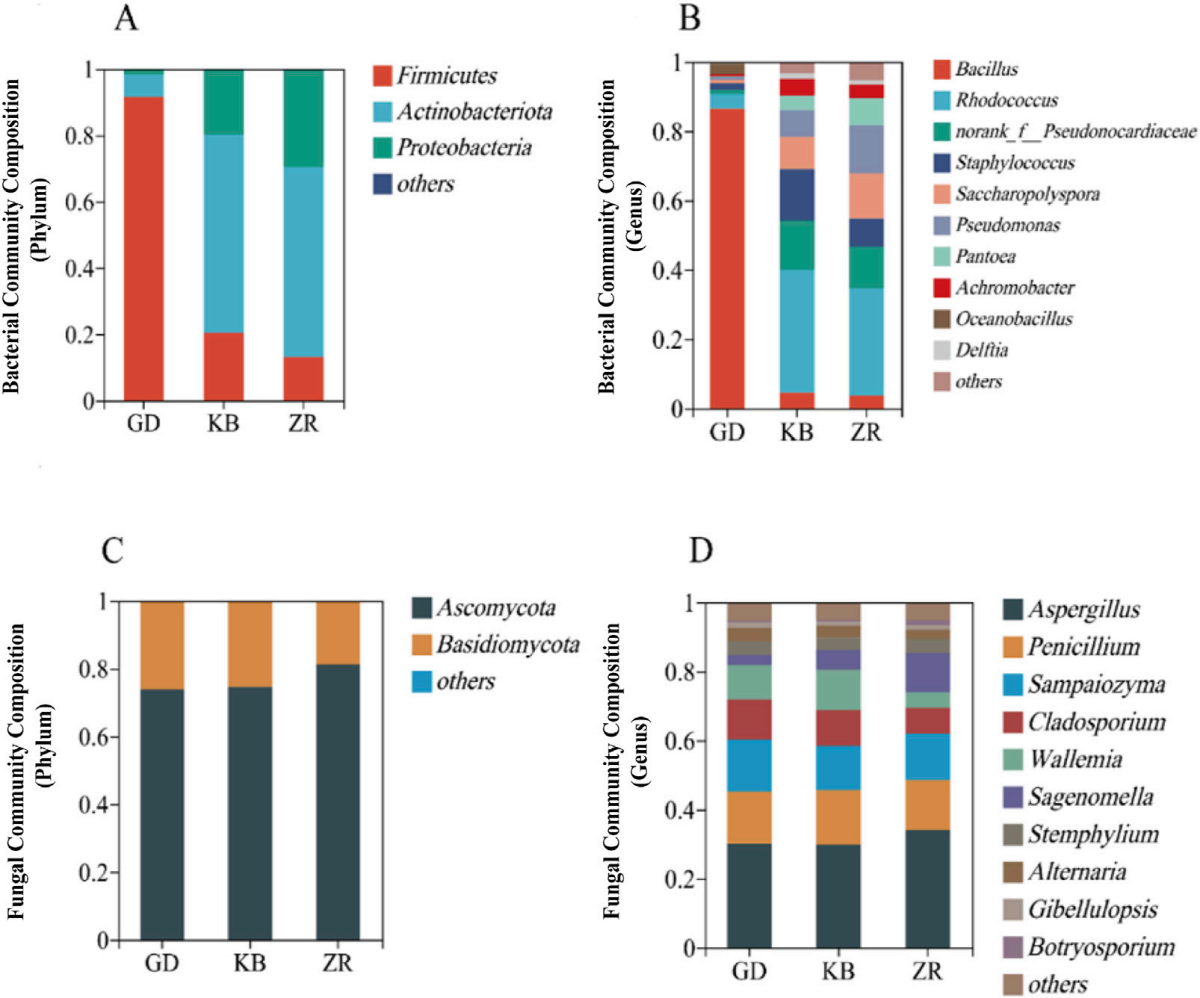
Community structure of bacteria and fungi in fermented cigar leaves. **(A)** Bacteria phylum level; **(B)** bacteria genus level; **(C)** fungal phylum level; **(D)** fungal genus level.

At the phylum level, the top four bacterial phyla were *Firmicutes*, *Actinobacteriota*, *Proteobacteria*, and *Chloroflexi*. At the genus level, the top four were *Bacillus*, *Rhodococcus*, *norank_f__Pseudonocardiaceae*, and *Staphylococcus*. To further investigate the impact of the added functional microorganism *Bacillus altitudinis* on microbial community structure, community composition was analyzed at both the phylum and genus levels (Figure 3).

At the phylum level, *Firmicutes* and *Ascomycota* were dominant in the GD group, whereas *Actinobacteria* and *Ascomycota* dominated in the KB and ZR groups. Compared to the other two groups, the GD group exhibited an increase in *Firmicutes* and a decrease in *Actinobacteria* among bacteria, while in fungi, *Ascomycota* decreased and *Basidiomycota* showed an increasing trend. At the genus level, the GD group showed elevated abundances of *Oceanobacillus* and *Bacillus*, while *Rhodococcus*, S*taphylococcus*, and *Achromobacter* decreased. For fungi, *Sampaiozyma*, *Cladosporium*, and *Wallemia* increased, whereas *Aspergillus* and *Sagenomella* declined. Notably, *Bacillus* became the dominant bacterial genus in the GD group, significantly altering the bacterial community composition of the fermented tobacco leaves (Zhou et al., 2024).

**FIGURE 4:**
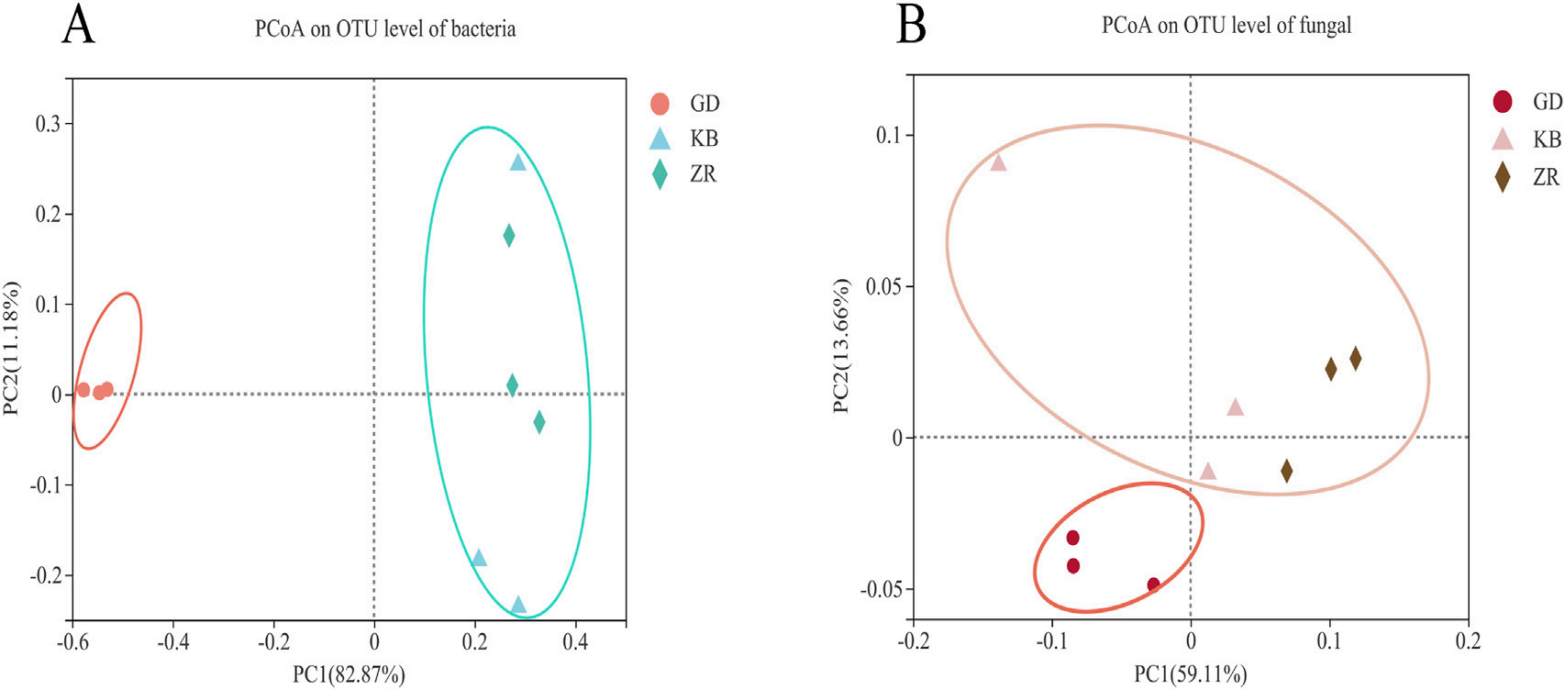
Principal coordinate analysis of bacteria **(A)** and fungi **(B)** in enhanced fermentation cigar leaves.

PCoA was conducted to compare the microbial community composition of CTLs across different fermentation groups (Figure 4). For bacterial communities, the variances explained by PC1 and PC2 were 82.87% and 11.18%, respectively, while for fungal communities, PC1 and PC2 accounted for 59.11% and 13.66% of the variance. The results indicated that both bacterial and fungal communities in the GD group were significantly distinct from those in the KB and ZR groups. In contrast, the ZR and KB groups clustered closely together, reflecting similar microbial community structures. These dimensionality reduction results demonstrate that the addition of the functional strain *Bacillus altitudinis* exerted a significant impact on the composition of microbial communities during cigar tobacco fermentation. Both bacterial and fungal communities in the enhanced fermentation group (GD) exhibited marked differences compared to the other groups.

### 3.4 Correlation between microoganisms and volatile compounds

**FIGURE 5:**
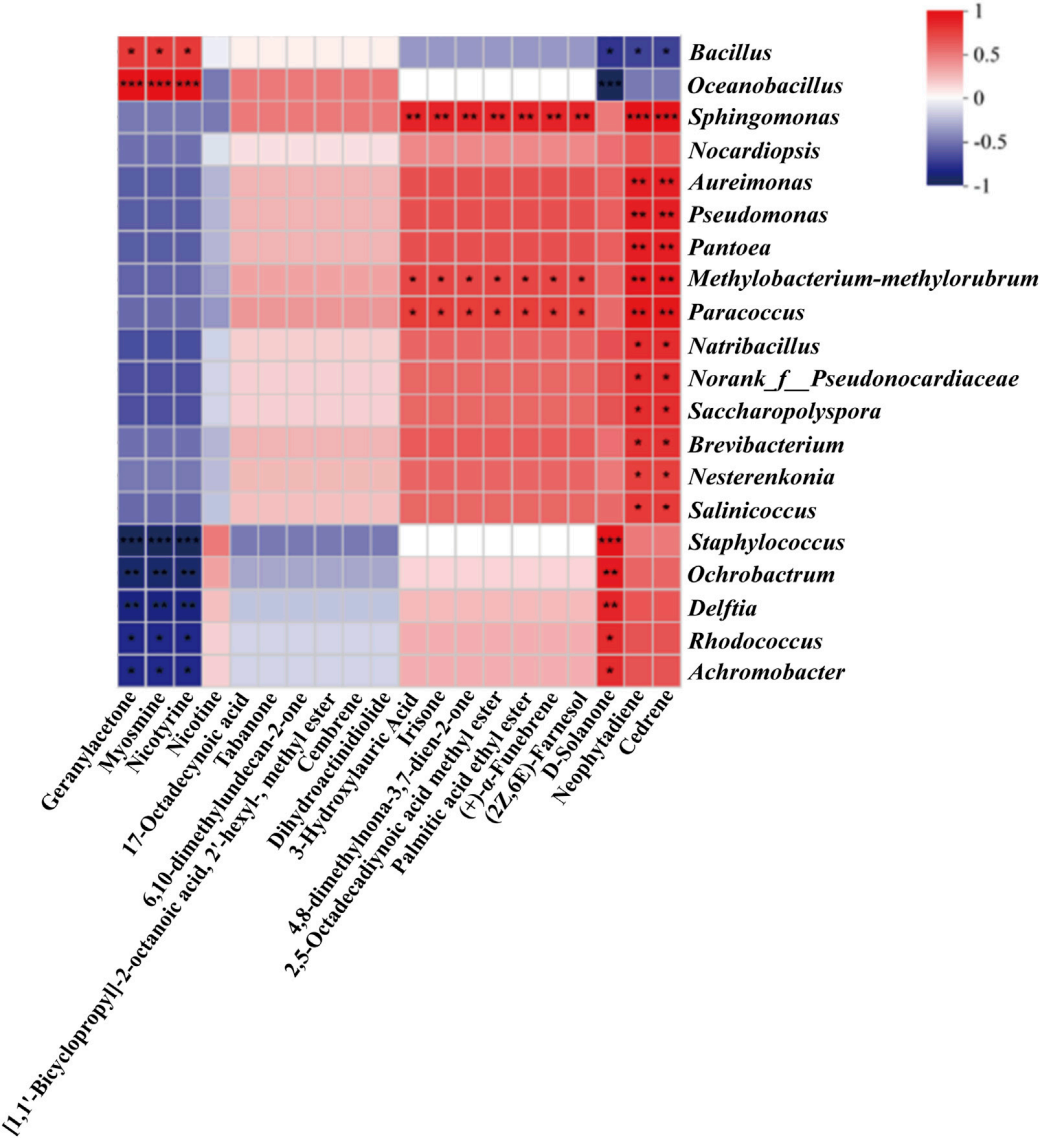
Heatmap of correlation between volatile metabolites and main microorganisms in CTLs with enhanced fermentation (|ρ | > 0.5, p-value <0.05, *p < 0.05, **p < 0.01, ***p < 0.001).

As the functional microorganism introduced in this study is a bacterium, the correlation network between bacterial communities and volatile metabolites was specifically analyzed, aiming to facilitate the future identification of beneficial microbes in cigar fermentation. A total of 20 representative volatile metabolites and the top 20 most abundant bacterial genera in the fermentation system were selected for correlation analysis (Figure 5).

First, nicotine degradation products such as myosmine and nicotyrine were significantly correlated with *Bacillus* and *Oceanobacillus* (Song et al., 2024). Geranylacetone and solanone were strongly associated with *Oceanobacillus*, *Staphylococcus*, *Ochrobactrum*, *Delftia*, *Rhodococcus*, and *Achromobacter*. Metabolites such as (±)-3-hydroxydodecanoic acid, β-ionone, nootkatone, methyl 2,5-octadecadiynoate, ethyl palmitate, (+)-α-cedrene, and 2Z,6E-farnesol exhibited significant correlations with *Sphingomonas* and *Methylobacterium-Methylorubrum*. Notably, neophytadiene and cedrene showed significant associations with 13 bacterial genera. Excess alkaloids in cigars not only impart bitterness and harshness during smoking but also pose health risks (Jia et al., 2023). It is widely accepted that alkaloid degradation reduces leaf harshness, resulting in a smoother and milder flavor profile (Xue et al., 2023), thus improving cigar tobacco quality. Correlation analysis confirmed the potential of microbial nicotine degradation. For instance, the nicotine degradation products myosmine and nicotyrine were significantly associated with *Bacillus* and *Oceanobacillus*—with the enhanced fermentation system specifically supplemented with *Bacillus altitudinis*, a member of the *Bacillus* genus. Additionally, solanone-related compounds are reported to play a key role in reducing impurities and harshness while enhancing the richness, smoothness, aftertaste, sweetness, and cleanliness of cigar aroma (Popova et al., 2019; Zheng et al., 2022). These compounds showed strong correlations with *Staphylococcus*, *Delftia*, and *Rhodococcus*. Terpenoids, which are derived from the MVA pathway and structurally composed of isoprene (C5) units (Tian et al., 2018), are known to be involved in the biosynthesis of plant volatiles (Cai et al., 2014; Wei et al., 2018; Shi et al., 2019; Czajka et al., 2020; Tang et al., 2022) and contribute unique aromas to cigars (Laureati et al., 2020; Song et al., 2020; Chen et al., 2022). These include compounds such as cedrene, neophytadiene, (+)-α-cedrene, and 2Z,6E-farnesol. Significant correlations were observed between terpenoids and genera including *Pantoea*, *Pseudomonas*, and *Sphingomonas*. Of particular note, *Sphingomonas* was significantly associated with multiple terpenoids and ketone-aldehyde compounds, including β-ionone, cedrene, and neophytadiene.

These findings suggest that further optimization of the bacterial community structure in cigar tobacco could enhance the content of aroma-contributing compounds. This highlights a promising direction for the discovery of beneficial microbes in cigar fermentation.

### 3.5 Sensory evaluation

**FIGURE 6:**
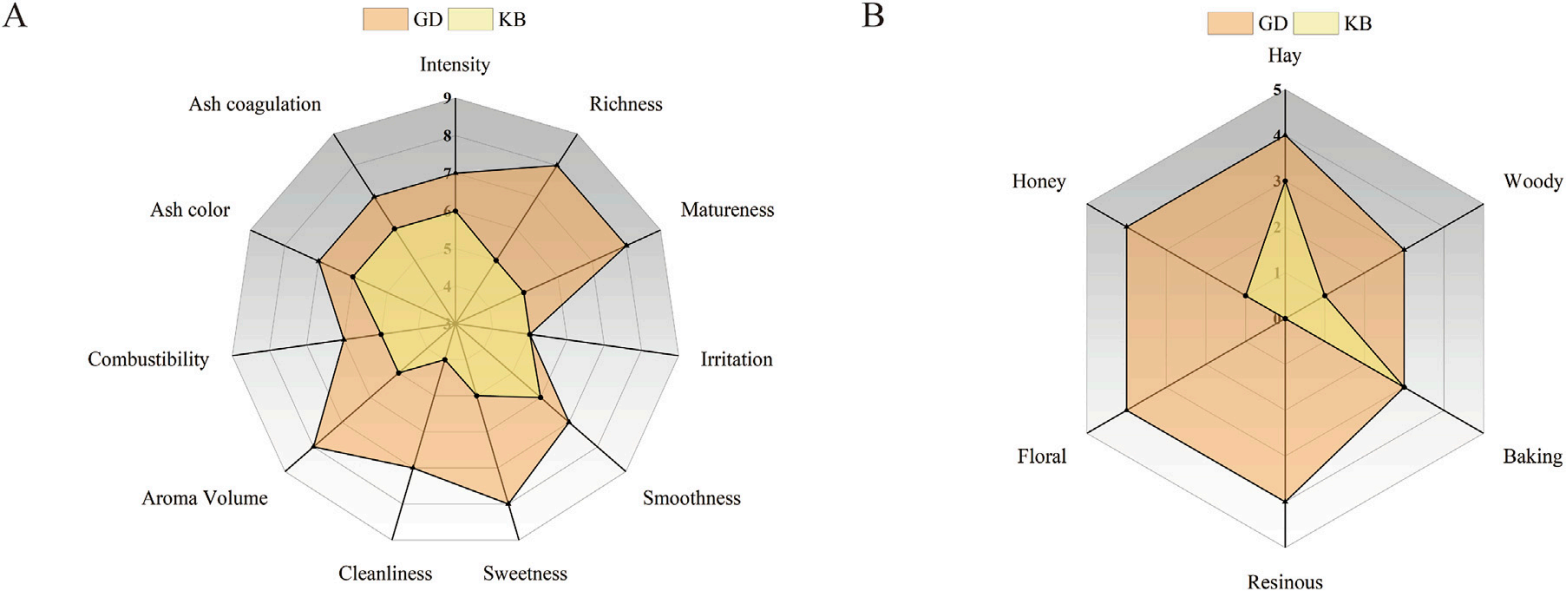
Radar plots of sensory evaluation of CTLs subjected to water fermentation and *Bacillus altitudinis*-enhanced fermentation. **(A)** Quality characteristics; **(B)** Flavor attributes. GD represents the enhanced fermentation group, and KB represents the control group.

Sensory evaluation is generally considered the most effective approach for assessing the fermentation performance of CTLs and the overall quality of cigars. To verify whether the isolated *Bacillus altitudinis* strain could improve CTLs quality through fermentation, sensory analysis was conducted on CTLs subjected to *Bacillus altitudinis*-enhanced fermentation, with water-fermented CTLs serving as the control. As shown in Figure 6, the total sensory scores of CTLs in the enhanced fermentation group were higher than those of the control group. Specifically, Figure 6A indicates that *Bacillus altitudinis* improved the cleanliness and maturity of CTLs while reducing irritation and off-flavors. In terms of aroma intensity, the fermentation process led to a richer, more mellow, and more harmonious aroma profile. Regarding aroma characteristics (Figure 6B), *Bacillus altitudinis* enhanced floral, honey-sweet, hay-like, and resinous notes in the CTLs. These sensory evaluation results were consistent with the volatile flavor compound analysis, confirming that *Bacillus altitudinis*-enhanced fermentation can effectively improve the quality of CTLs.

## 4 Discussion

Through functional strain-enhanced fermentation, the flavor and smoking characteristics of CTLs can be significantly improved. In order to enhance cigar quality, reduce the nicotine content of CTLs, and optimize cigar flavor, this study isolated a strain of *Bacillus altitudinis* from high-quality CTLs produced in the Shifang region. This strain was used to enhance the fermentation of CTLs. GC-MS analysis of the flavor compounds after enhanced fermentation revealed a 31.6% reduction in tobacco alkaloid content, a 13.4% increase in aldehyde and ketone levels, a 64.3% increase in terpenoid compounds, significant increases in acid content, and a slight increase in ester compounds. Ten new compounds with desirable flavor characteristics were identified, including linalool, geraniol, (±)-3-hydroxylauric acid, and citrus ketone. The aroma profile analysis showed enhanced floral, honey-like, hay, and resinous notes in CTLs. Regarding smoking characteristics, the effective degradation of nitrogen-containing compounds improved smoothness, maturity, and reduced irritants and off-flavors, making the aroma softer and more refined. Sensory evaluation results aligned with the analysis of volatile flavor compounds, confirming that the *Bacillus altitudinis* strain could improve the quality of CTLs through fermentation.

The fermentation of CTLs is a synergistic outcome of microbial community interactions. Nicotine degradation is closely associated with the genera *Bacillus* and *Oceanobacillus* (Song et al., 2024). The formation of geranylacetone and solanone is significantly correlated with *Staphylococcus*, *Ochrobactrum*, and *Achromobacter*, while (±)-3-hydroxydodecanoic acid, β-ionone, and 2Z,6E-farnesol are strongly associated with *Sphingomonas* and *Methylobacterium-Methylorubrum*. In the present study, the addition of *Bacillus altitudinis* significantly altered the microbial community structure of CTLs, as confirmed by PCoA. Specifically, bacterial community changes were characterized by increased abundances of *Oceanobacillus* and *Bacillus*, and decreased abundances of *Rhodococcus*, *Staphylococcus*, and *Achromobacter*. Within the fungal community, the relative abundances of *Sampaiozyma*, *Cladosporium*, and *Wallemia* increased, while those of *Aspergillus* and *Sagenomella* decreased; however, these changes in the fungal community were not statistically significant.

**FIGURE 7:**
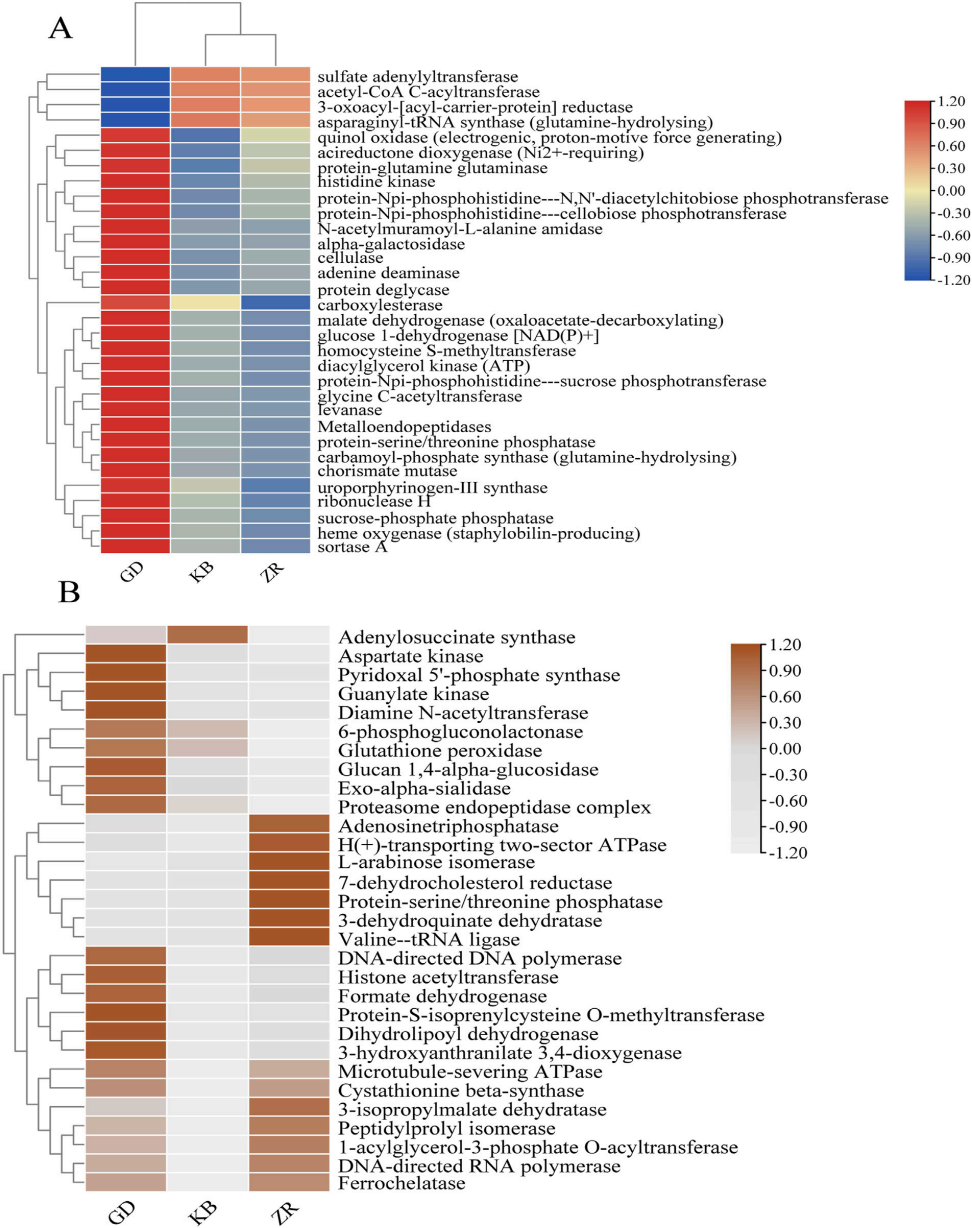
Systematic correlation analysis of genomic, functional, and enzymatic information was performed based on the KEGG database. **(A)** Variation in bacterial enzyme abundance across the GD, KB, and ZR groups; **(B)** Variation in fungal enzyme abundance across the GD, KB, and ZR groups (Supplementary Table S2 for detailed data).

To further investigate the enzymatic-level effects of fermentation enhanced by *Bacillus altitudinis*, based on KEGG database annotations, the relationships among genomic, functional, and enzymatic features were analyzed, and enzyme abundance was quantified accordingly. Following the addition of *Bacillus altitudinis*, significant shifts in the enzymatic profile of the microbial community in CTLs were observed (Figure 7). Interestingly, although the fungal community showed no significant changes in species abundance or diversity following enhanced fermentation, functional annotation of fungal genes revealed marked differences in enzyme expression compared to the control group. This suggests that the addition of *Bacillus altitudinis*, while not significantly altering the composition of the fungal community, influenced its gene expression and metabolic pathways, thereby resulting in substantial shifts in the fungal enzymatic expression profile. Specifically, enzymes associated with protein degradation and amino acid metabolism showed markedly increased abundances, including acetyl-CoA C-acetyltransferase, acireductone dioxygenase (Ni^2+^-requiring), protein-glutamine glutaminase, glycine C-acetyltransferase, metalloendopeptidases, and aspartate kinase. Among them, acetyl-CoA C-acetyltransferase is involved in the degradation of valine, leucine, isoleucine, and lysine, as well as tryptophan metabolism; acireductone dioxygenase (Ni^2+^-requiring) participates in cysteine and methionine metabolism (Al-Mjeni et al., 2002); protein-glutamine glutaminase is related to glutamine hydrolysis (Kikuchi et al., 1971); glycine C-acetyltransferase and aspartate kinase are associated with glycine (Schmidt et al., 2001) and aspartate metabolism (Chassagnole et al., 2001; Curien et al., 2005), respectively. Metalloendopeptidases catalyze peptide bond hydrolysis in proteins (Bond and Beynon, 1995). These findings further support earlier experimental evidence that this functional strain is capable of degrading nitrogen-containing compounds such as proteins. Additionally, the enhanced fermentation process showed increased abundance of several enzymes involved in carbohydrate metabolism, such as cellulase, protein-Npi-phosphohistidine---sucrose phosphotransferase, levanase, and alpha-galactosidase, which are associated with the degradation of polysaccharides including glucans, fructans, and cellulose. A notable increase was also observed in malate dehydrogenase (oxaloacetate-decarboxylating), an enzyme involved in pyruvate metabolism (de Lorenzo et al., 2024).

**FIGURE 8:**
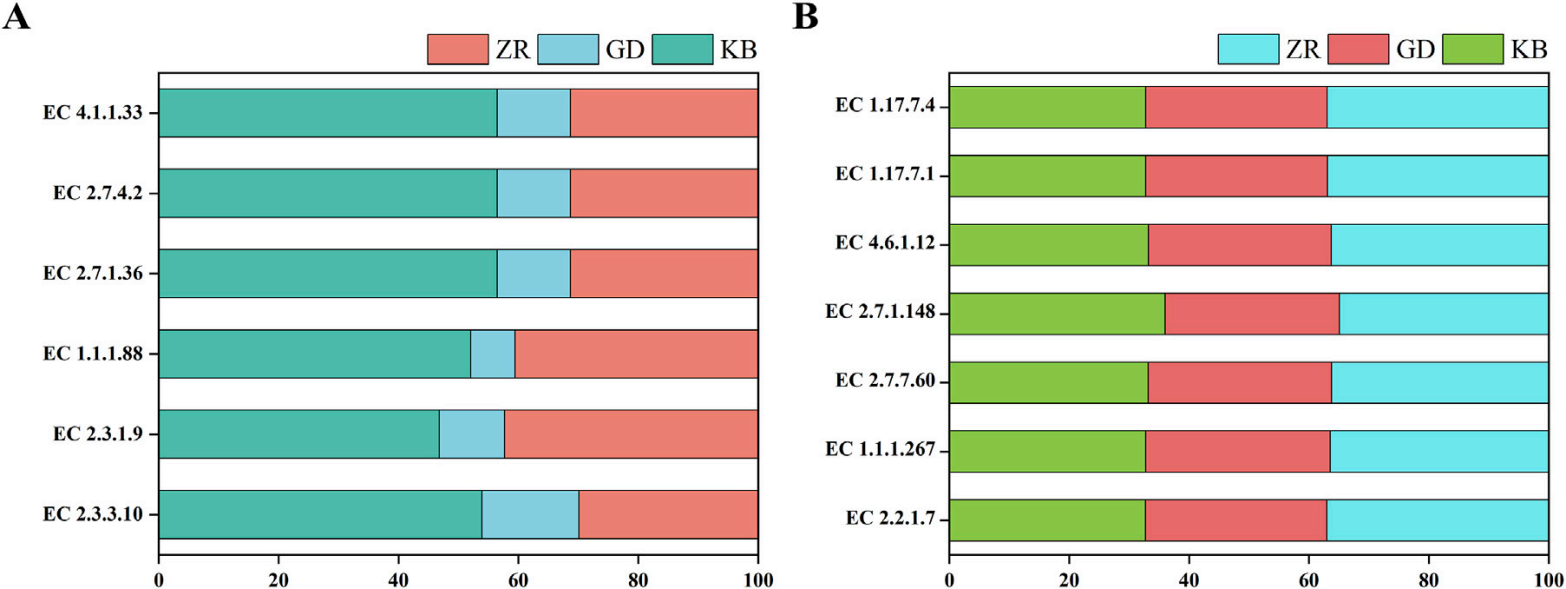
Based on KEGG pathway analysis, the variations in key enzymes related to terpenoid biosynthesis were compared among groups. **(A)** Changes in MVA pathway-related enzyme sys-tems in GD, KB, and ZR groups; **(B)** Changes in MEP pathway-related enzyme systems in GD, KB, and ZR groups (Supplementary Table S3 for detailed data).

These enzymatic changes may explain the observed enhancement in the production of volatile flavor compounds during fermentation. The addition of *Bacillus altitudinis* effectively accelerated the degradation of nitrogenous and carbohydrate substances, resulting in increased availability of small molecules for microbial metabolism. This in turn promoted active microbial metabolism and increased activity of terpene synthases (TPS), which are essential for the biosynthesis of volatile flavor compounds, most of which are derived from carotenoid metabolism (Liu et al., 2024). As illustrated, glucose and fructose are metabolized via glycolysis to yield acetyl-CoA and pyruvate, the starting substrates for the MVA and MEP pathways, respectively. These pathways lead to the formation of isopentenyl diphosphate (IPP) and dimethylallyl diphosphate (DMAPP), key precursors in carotenoid biosynthesis. Interestingly, the abundance of key enzymes in the MVA pathway decreased markedly after the addition of *Bacillus altitudinis*, while those in the MEP pathway remained unchanged, suggesting that carotenoid precursor biosynthesis primarily proceeds through the MEP pathway during enhanced fermentation (Figure 8).

**FIGURE 9:**
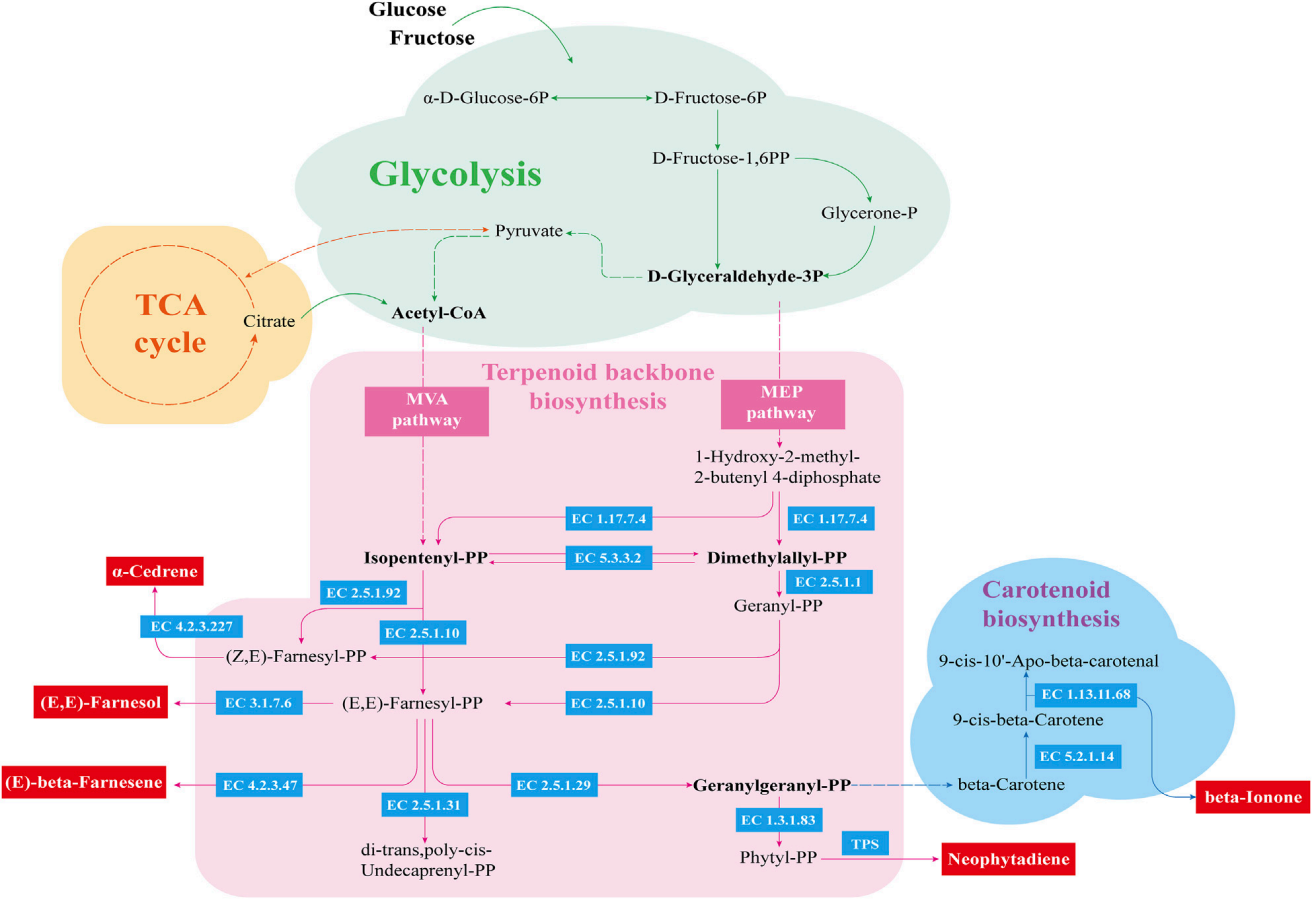
Metabolic network of major volatile flavor compounds. The green area indicates the glycolysis pathway, the pink area represents the synthesis pathway of terpene skeleton com-pounds, and the blue area corresponds to the carotenoid metabolic pathway. Enzyme codes shown in blue boxes are derived from the KEGG database.

To provide a more comprehensive analysis of the synthesis mechanisms of volatile flavor compounds, the metabolic pathway was visualized based on the KEGG database (Figure 9). The synthesis of carotenoid precursors involves multiple enzymes secreted by microorganisms. Geranylgeranyl diphosphate synthase (EC 2.5.1.1) is a critical enzyme that catalyzes the condensation of IPP and DMAPP to form geranylgeranyl diphosphate (GGPP), a central precursor in carotenoid biosynthesis. In addition to serving as substrates for carotenoids, IPP and DMAPP also lead to the production of mono- and sesquiterpenes such as cedrene (Wang et al., 2024). Carotenoid metabolism further yields various aromatic ketones, including β-ionone, solanone, and geranylacetone (Meléndez-Martínez et al., 2023), all of which contribute significantly to tobacco aroma. Based on the metabolite profiles and metabolic pathway analysis, it is postulated that the enhanced fermentation process activated additional terpene metabolic branches, leading to the biosynthesis of new terpenoid compounds such as (+)-α-cedrol and 2Z,6E-farnesol, which were not detected in the ZR and KB groups. Therefore, precise regulation of IPP and DMAPP metabolic flux may be a key strategy for improving aroma quality (Rasouli et al., 2018).

## 5 Conclusion

In conclusion, *Bacillus altitudinis* isolated from CTLs plays a key role in the fermentation process of cigar tobacco by regulating the tobacco microbiota, metabolic pathways, and improving aroma. *Bacillus altitudinis* can enhance the substrate availability on the surface of CTLs by degrading proteins and other macromolecules, activate carotenoid metabolic pathways, and promote the synthesis of flavor compounds. In the regulation of chemical composition, it can reduce the total amount of tobacco alkaloids and promote the synthesis of terpenes and other flavor compounds. In the regulation of the tobacco microbiota, it promotes the accumulation of aldehydes and ketones by selectively modulating key genera such as *Oceanobacillus* and *Staphylococcus*. Future research could further elucidate the coupling mechanism between the strain’s genomic functional modules and metabolic pathways, and explore its potential for large-scale industrial fermentation applications.

## Data Availability

The data presented in the study are deposited in NCBI Sequence Read Archive (SRA, https://www.ncbi.nlm.nih.gov/sra/PRJNA824254) database under the BioProject accession number PRJNA824254.
