# Supplementary material for: Isolation and evaluation of a Bacillus altitudinis strain to improve cigar tobacco leaves fermentation
Source: Front Bioeng Biotechnol. 2025 Aug 12;13:1636506. doi: 10.3389/fbioe.2025.1636506 (PMC12378408; doi:10.3389/fbioe.2025.1636506)
Supplement: Supplementary file 1 [file DataSheet1.zip › supplementary.docx]

Table S1. Content of volatile substances in cigar tobacco of Shifang binder leaf by *Bacillus altitudinis* enhanced fermentation

| Category | CAS | Compound Name | Content in KB Group (µg/Kg) | Content in GD Group (µg/Kg) | Content in ZR Group (µg/Kg) |
| --- | --- | --- | --- | --- | --- |
| Tobacco  Alkaloids | 23950-04-1 | Nicotine | 85006.70±382.34 | 57533.07±1836.31 | 95772.28±189.23 |
|  | 532-12-7 | Myosmine | 487.44±4.34 | 385.28±54.84 | 0.00 |
|  | 487-19-4 | Nornicotine | 2443.84±100.53 | 2222.40±138.85 | 296.14±5.43 |
| Total | | | 87937.99±463.98 | 60140.75±2029.98 | 96068.42±194.66 |
| Terpenes | 504-96-1 | Neophytadiene | 17901.94±46.42 | 29360.02±835.02 | 24849.2±4160.29 |
|  | 50894-66-1 | (+)-α-Cedrene | 0.00 | 120.35±0.00 | 0.00 |
|  | 3790-71-4 | 2Z,6E-Farnesol | 0.00 | 34.18±0.00 | 0.00 |
|  | 11028-42-5 | Cedrolene | 0.00 | 120.15±27.60 | 113.35±2.10 |
|  | 1898-13-1 | β-Selinene | 455.18±12.61 | 532.48±3.01 | 19.97±0.37 |
| Total | | | 18357.12±55.57 | 30167.19±804.48 | 24982.52±4162.76 |
| Esters | 103-45-7 | Phenethyl Acetate | 20000±0.00 | 20000±0.00 | 20000±0.00 |
|  | 628-97-7 | Ethyl Palmitate | 0.00 | 75.04±4.31 | 0.00 |
|  | 17092-92-1 | Dihydroactinidiolide | 941.91±6.89 | 951.05±21.88 | 356.69±79.63 |
|  | 56687-68-4 | 2-Hexyl-1,1-bicyclopropane-2-octanoate | 52.71±3.35 | 212.48±67.91 | 0.00 |
|  | 57156-91-9 | Methyl 2,5-octadecadynoate | 0.00 | 147.72±0.00 | 0.00 |
| Total | | | 20994.63±10.28 | 21386.29±82.50 | 20356.69±79.63 |
| Aldehydes  and Ketone | 3796-70-1 | Damascone | 4796.34±199.72 | 4675.78±264.70 | 2656.55±53.10 |
|  | 54868-48-3 | Solanone | 853.41±38.47 | 1015.25±65.34 | 1199.12±5.95 |
|  | 1604-34-8 | Hexahydro-β-ionone | 473.91±3.72 | 576.39±50.99 | 0.00 |

| Category | CAS | Compound Name | Content in KB Group (µg/Kg) | Content in GD Group (µg/Kg) | Content in ZR Group (µg/Kg) |
| --- | --- | --- | --- | --- | --- |
| Aldehydes  and Ketone | 817-88-9 | Citral | 0.00 | 309.80±1.98 | 0.00 |
|  | 14901-07-6 | β-Ionone | 0.00 | 115.13±20.71 | 0.00 |
|  | 13215-88-8 | Jasmone | 740.31±8.94 | 1090.08±63.72 | 188.91±58.74 |
| Total | | | 6863.96±247.40 | 7782.34±397.94 | 4044.58±117.79 |
| Acids | 34450-18-5 | 17-Octadecy-noic acid | 110.93±2.00 | 134.39±1.34 | 0.00 |
|  | 1883-13-2 | (±)-3-Hydro-xylauric acid | 0.00 | 315.62±0.00 | 0.00 |
| Total | | | 110.93±2.00 | 450.01±1.34 | 0.00±0.00 |

Table S2. Comparative Abundance of Key Metabolic Enzymes Across ZR, GD, and KB Groups.

| Enzyme ID | Enzyme Name | Enzyme Abundance | | |
| --- | --- | --- | --- | --- |
|  |  | KB group | GD group | ZR group |
| 2.7.13.3 | histidine kinase | 39372 | 61546 | 43437 |
| 3.5.1.28 | N-acetylmuramoyl-L-alanine amidase | 7594 | 22336 | 7656 |
| 3.4.24.- | Metalloendopeptidases | 12447 | 16278 | 12059 |
| 1.1.1.100 | 3-oxoacyl-[acyl-carrier-protein] reductase | 29806 | 15050 | 28079 |
| 3.1.3.24 | sucrose-phosphate phosphatase | 6629 | 13929 | 5674 |
| 6.3.5.6 | asparaginyl-tRNA synthase (glutamine-hydrolysing) | 22966 | 12667 | 21493 |
| 3.1.1.1 | carboxylesterase | 7920 | 9092 | 6792 |
| 3.1.26.4 | ribonuclease H | 5636 | 7280 | 5204 |
| 7.1.1.7 | quinol oxidase (electrogenic, proton-motive force generating) | 6512 | 7255 | 6793 |
| 4.2.1.75 | uroporphyrinogen-III synthase | 5860 | 7209 | 5346 |
| 6.3.5.5 | carbamoyl-phosphate synthase (glutamine-hydrolysing) | 4961 | 7202 | 4781 |
| 2.7.1.107 | diacylglycerol kinase (ATP) | 4254 | 7079 | 3961 |
| 5.4.99.5 | chorismate mutase | 4693 | 7075 | 4497 |
| 1.14.99.48 | heme oxygenase (staphylobilin-producing) | 4523 | 7044 | 4089 |
| 1.1.1.38 | malate dehydrogenase (oxaloacetate-decarboxylating) | 3069 | 6889 | 2661 |
| 1.1.1.47 | glucose 1-dehydrogenase [NAD(P)+] | 946 | 3382 | 752 |
| 2.3.1.29 | glycine C-acetyltransferase | 757 | 3365 | 688 |
| 3.4.22.70 | sortase A | 670 | 3365 | 458 |
| 3.2.1.4 | cellulase | 398 | 3352 | 508 |
| 2.1.1.10 | homocysteine S-methyltransferase | 735 | 3328 | 561 |
| 1.13.11.53 | acireductone dioxygenase (Ni2+-requiring) | 895 | 3320 | 1294 |
| 3.5.4.2 | adenine deaminase | 225 | 3305 | 288 |
| 3.2.1.22 | alpha-galactosidase | 242 | 3264 | 262 |
| 3.2.1.65 | levanase | 173 | 3258 | 142 |
| 2.7.7.4 | sulfate adenylyltransferase | 13268 | 3217 | 12452 |
| 2.3.1.16 | acetyl-CoA C-acyltransferase | 9945 | 2868 | 9193 |
| 2.7.1.196 | protein-Npi-phosphohistidine---N,N'-diacetylchitobiose phosphotransferase | 820 | 5000 | 1130 |
| 2.7.1.205 | protein-Npi-phosphohistidine---cellobiose phosphotransferase | 820 | 5000 | 1130 |
| 2.7.1.211 | protein-Npi-phosphohistidine---sucrose phosphotransferase | 804 | 4953 | 603 |
| 3.5.1.124 | protein deglycase | 1952 | 3611 | 2038 |
| 3.5.1.44 | protein-glutamine glutaminase | 2097 | 3508 | 2452 |
| 3.1.3.16 | protein-serine/threonine phosphatase | 3391 | 5366 | 3217 |
|  |  |  |  |  |
| 3.2.1.3 | Glucan 1,4-alpha-glucosidase | 2782893.9 | 2676613.2 | 2613684.2 |
| 3.6.1.3 | Adenosinetriphosphatase | 2624598.1 | 2603204.1 | 2653078.8 |
| 5.3.1.4 | L-arabinose isomerase | 2160358.1 | 2183114.3 | 2322142.1 |
| 2.7.7.6 | DNA-directed RNA polymerase | 1882466.8 | 1851755.1 | 1889339.5 |
| 5.2.1.8 | Peptidylprolyl isomerase | 1646667.4 | 1641830.7 | 1648264.6 |
| 3.2.1.18 | Exo-alpha-sialidase | 1596848.7 | 1569940.2 | 1545794.9 |
| 2.7.7.7 | DNA-directed DNA polymerase | 1583564.3 | 1578890 | 1581319.8 |
| 3.4.25.1 | Proteasome endopeptidase complex | 1299564.8 | 1289927.2 | 1278206.6 |
| 2.3.1.48 | Histone acetyltransferase | 1295809.7 | 1264757.9 | 1277554.4 |
| 3.6.3.14 | H(+)-transporting two-sector ATPase | 1227950.9 | 1221873 | 1238413.9 |
| 3.1.3.16 | Protein-serine/threonine phosphatase | 1216839.2 | 1216890.2 | 1224387.2 |
| 1.8.1.4 | Dihydrolipoyl dehydrogenase | 60227.06 | 60095.84 | 60129.79 |
| 6.3.4.4 | Adenylosuccinate synthase | 60095.71 | 60183.53 | 59971.12 |
| 4.2.1.10 | 3-dehydroquinate dehydratase | 60058.45 | 60004.2 | 63844.9 |
| 1.3.1.21 | 7-dehydrocholesterol reductase | 60051.69 | 59661.93 | 63246.62 |
| 2.7.2.4 | Aspartate kinase | 60040.71 | 59949.52 | 59922.46 |
| 2.3.1.51 | 1-acylglycerol-3-phosphate O-acyltransferase | 60021.7 | 59801.19 | 60087.45 |
| 2.7.4.8 | Guanylate kinase | 60005.72 | 59913.52 | 59896.78 |
| 1.13.11.6 | 3-hydroxyanthranilate 3,4-dioxygenase | 59994.76 | 59861.48 | 59900.94 |
| 2.3.1.57 | Diamine N-acetyltransferase | 59985.73 | 59892.19 | 59877.44 |
| 3.1.1.31 | 6-phosphogluconolactonase | 59975.37 | 59923.51 | 59799.78 |
| 1.11.1.9 | Glutathione peroxidase | 59974.7 | 59920.84 | 59799.45 |
| 1.4.3.5 | Pyridoxal 5'-phosphate synthase | 59941.05 | 59860.55 | 59853.12 |
| 1.2.1.2 | Formate dehydrogenase | 59852.42 | 59765.17 | 59805.93 |
| 4.2.1.33 | 3-isopropylmalate dehydratase | 59821.77 | 59679.51 | 59909.27 |
| 4.99.1.1 | Ferrochelatase | 59798.39 | 59705.18 | 59810.46 |
| 6.1.1.9 | Valine--tRNA ligase | 59760.38 | 59760.16 | 59794.46 |
| 3.6.4.3 | Microtubule-severing ATPase | 59728.7 | 59685.78 | 59720.79 |
| 4.2.1.22 | Cystathionine beta-synthase | 59663.71 | 59626.49 | 59661.46 |
| 2.1.1.100 | Protein-S-isoprenylcysteine O-methyltransferase | 59641.04 | 59622.48 | 59625.43 |

Table S3. Key Enzyme Systems in the MVA and MEP Biosynthetic Pathways.

| Enzyme ID | Enzyme Name | Enzyme Abundance | | |
| --- | --- | --- | --- | --- |
|  |  | KB group | GD group | ZR group |
| MVA pathway | | | | |
| 2.3.3.10 | hydroxymethylglutaryl-CoA synthase | 449 | 135 | 249 |
| 2.3.1.9 | acetyl-CoA C-acetyltransferase | 28638 | 6664 | 25851 |
| 1.1.1.88 | hydroxymethylglutaryl-CoA reductase | 1759 | 249 | 1371 |
| 2.7.1.36 | mevalonate kinase | 449 | 97 | 249 |
| 2.7.4.2 | phosphomevalonate kinase | 449 | 97 | 249 |
| 4.1.1.33 | diphosphomevalonate decarboxylase | 449 | 97 | 249 |
| MEP pathway | | | | |
| 2.2.1.7 | 1-deoxy-D-xylulose-5-phosphate synthase | 1994 | 1845 | 2254 |
| 1.1.1.267 | 1-deoxy-D-xylulose-5-phosphate reductoisomerase | 1959 | 1840 | 2182 |
| 2.7.7.60 | 2-C-methyl-D-erythritol 4-phosphate cytidylyltransferase | 2001 | 1843 | 2186 |
| 2.7.1.148 | 4-(cytidine 5'-diphospho)-2-C-methyl-D-erythritol kinase | 2395 | 1933 | 2321 |
| 4.6.1.12 | 2-C-methyl-D-erythritol 2,4-cyclodiphosphate synthase | 2006 | 1843 | 2192 |
| 1.17.7.1 | (E)-4-hydroxy-3-methylbut-2-enyl-diphosphate synthase (ferredoxin) | 1991 | 1844 | 2245 |
| 1.17.7.4 | 4-hydroxy-3-methylbut-2-en-1-yl diphosphate reductase | 1993 | 1844 | 2248 |


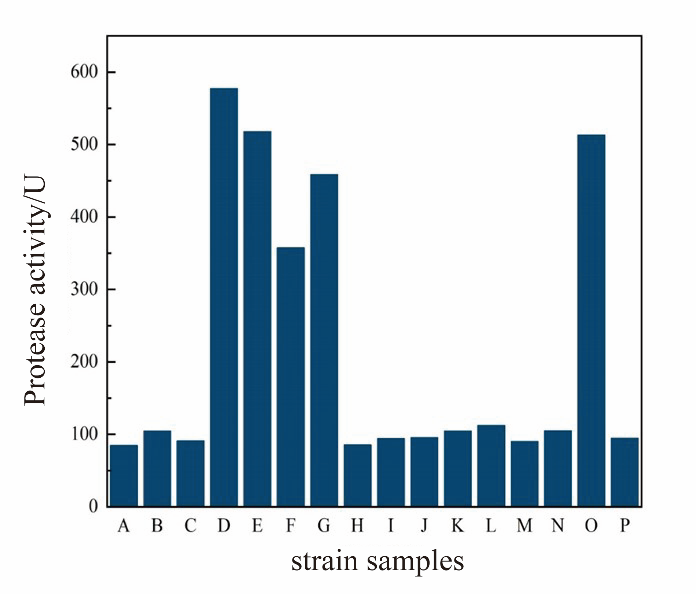


Figure S1. Determination of protease activity of 16 strains screened.


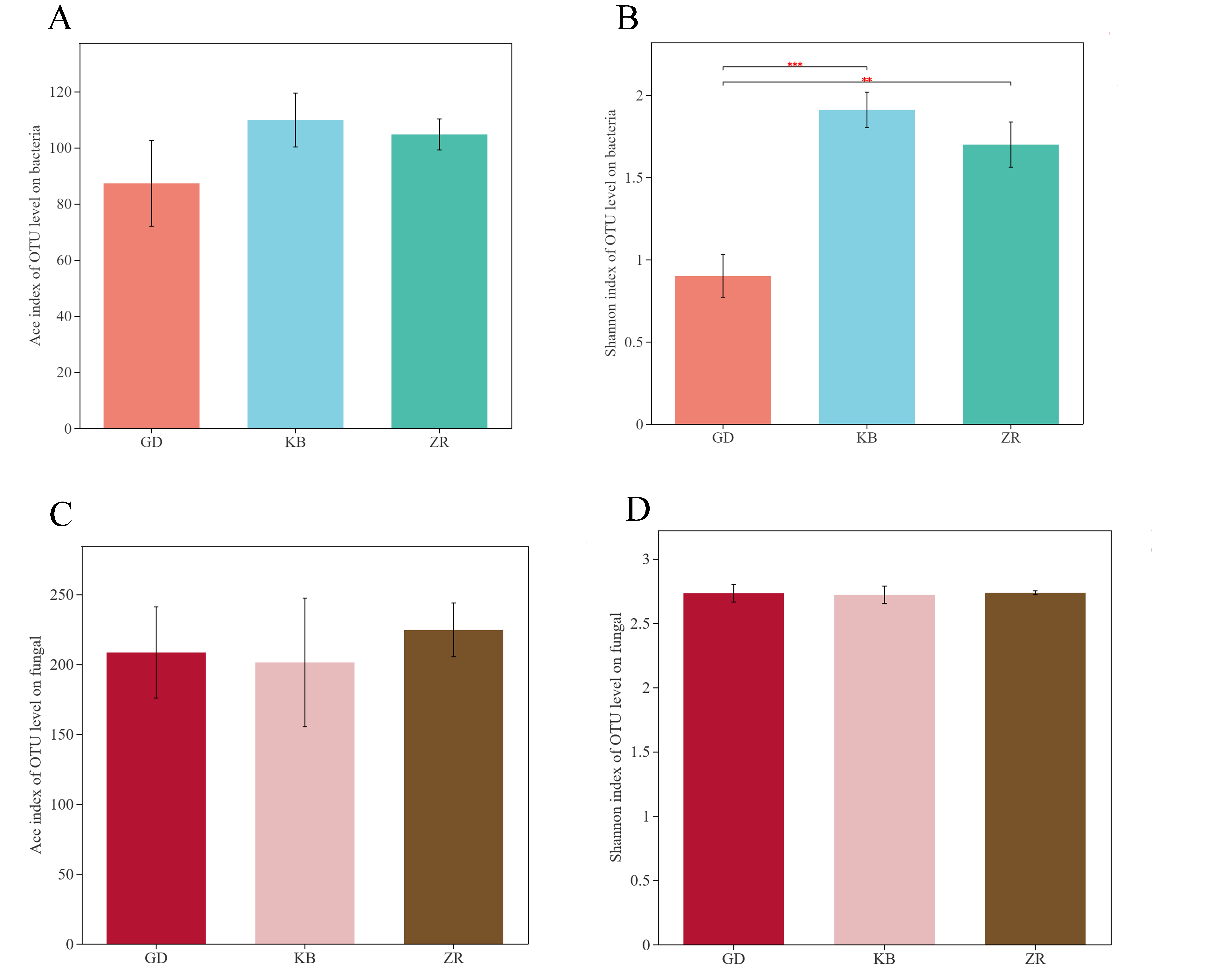


Figure S2. Enhanced fermented cigar leaves microbial diversity and richness.(A) bacterial richness; (B) Bacterial diversity; (C) fungal richness; (D) fungal diversity.
